# Robotic CT-guided out-of-plane needle insertion: comparison of angle accuracy with manual insertion in phantom and measurement of distance accuracy in animals

**DOI:** 10.1007/s00330-019-06477-1

**Published:** 2019-11-26

**Authors:** Toshiyuki Komaki, Takao Hiraki, Tetsushi Kamegawa, Takayuki Matsuno, Jun Sakurai, Ryutaro Matsuura, Takuya Yamaguchi, Takanori Sasaki, Toshiharu Mitsuhashi, Soichiro Okamoto, Mayu Uka, Yusuke Matsui, Toshihiro Iguchi, Hideo Gobara, Susumu Kanazawa

**Affiliations:** 1 Department of Radiology, Okayama University Medical School, 2-5-1 Shikatacho, Kitaku, Okayama, 700-8558 Japan; 2 Graduate School of Interdisciplinary Science and Engineering in Health Systems, Okayama University, 3-1-1 Tsushimanaka, Kitaku, Okayama, 700-8530 Japan; 3 Graduate School of Natural Science and Technology, Okayama University, 3-1-1 Tsushimanaka, Kitaku, Okayama, 700-8530 Japan; 4 Center for Innovative Clinical Medicine, Okayama University Hospital, 2-5-1 Shikatacho, Kitaku, Okayama, 700-8558 Japan; 5 Graduate School of Health Sciences, Okayama University Medical School, 2-5-1 Shikatacho, Kitaku, Okayama, 700-8558 Japan; 6 Division of Radiology, Department of Medical Technology, Okayama University Hospital, 2-5-1 Shikatacho, Kitaku, Okayama, 700-8558 Japan; 7 Collaborative Research Center for OMIC, Okayama University Graduate School of Medicine, Dentistry, and Pharmaceutical Sciences, 2-5-1 Shikatacho, Kitaku, Okayama, 700-8558 Japan; 8 Division of Medical Informatics, Okayama University Hospital, 2-5-1 Shikatacho, Kitaku, Okayama, 700-8558 Japan

**Keywords:** Robotics, Interventional radiology, Animal experiments

## Abstract

**Objectives:**

To evaluate the accuracy of robotic CT-guided out-of-plane needle insertion in phantom and animal experiments.

**Methods:**

A robotic system (Zerobot), developed at our institution, was used for needle insertion. In the phantom experiment, 12 robotic needle insertions into a phantom at various angles in the *XY* and *YZ* planes were performed, and the same insertions were manually performed freehand, as well as guided by a smartphone application (SmartPuncture). Angle errors were compared between the robotic and smartphone-guided manual insertions using Student’s *t* test. In the animal experiment, 6 robotic out-of-plane needle insertions toward targets of 1.0 mm in diameter placed in the kidneys and hip muscles of swine were performed, each with and without adjustment of needle orientation based on reconstructed CT images during insertion. Distance accuracy was calculated as the distance between the needle tip and the target center.

**Results:**

In the phantom experiment, the mean angle errors of the robotic, freehand manual, and smartphone-guided manual insertions were 0.4°, 7.0°, and 3.7° in the *XY* plane and 0.6°, 6.3°, and 0.6° in the *YZ* plane, respectively. Robotic insertions in the *XY* plane were significantly (*p* < 0.001) more accurate than smartphone-guided insertions. In the animal experiment, the overall mean distance accuracy of robotic insertions with and without adjustment of needle orientation was 2.5 mm and 5.0 mm, respectively.

**Conclusion:**

Robotic CT-guided out-of-plane needle insertions were more accurate than smartphone-guided manual insertions in the phantom and were also accurate in the in vivo procedure, particularly with adjustment during insertion.

**Key Points:**

• *Out-of-plane needle insertions performed using our robot were more accurate than smartphone-guided manual insertions in the phantom experiment and were also accurate in the in vivo procedure.*

• *In the phantom experiment, the mean angle errors of the robotic and smartphone-guided manual out-of-plane needle insertions were 0.4° and 3.7° in the XY plane (p < 0.001) and 0.6° and 0.6° in the YZ plane (p = 0.65), respectively.*

• *In the animal experiment, the overall mean distance accuracies of the robotic out-of-plane needle insertions with and without adjustments of needle orientation during insertion were 2.5 mm and 5.0 mm, respectively.*

**Electronic supplementary material:**

The online version of this article (10.1007/s00330-019-06477-1) contains supplementary material, which is available to authorized users.

## Introduction

Computed tomography (CT)-guided interventional procedures such as ablation and biopsy primarily comprise needle insertion into the lesion under CT guidance. Although needle insertion is generally performed in the axial CT plane (i.e., in-plane insertion), out-of-plane needle insertion is occasionally required to achieve an anatomically safer tract. For example, needle insertion into the hepatic dome of the liver and the renal upper pole along a craniocaudally oblique tract may avoid transthoracic insertion accompanied by risks of pneumothorax and hemothorax [1, 2]. However, such needle insertions with freehand manual techniques are generally challenging because it is difficult to set a needle at the angle required and to maintain it as such during insertion [3]. Further, adjustment of needle orientation based on CT images during insertion is also difficult, because the entire needle and the target are not observed in the same two-dimensional CT plane. Additionally, target movement due to respiration may make it even more difficult. Although multiplanar reconstructions may help confirm needle orientation, they require additional time and expertise.

We have been developing a robotic system to enable CT-guided needle insertion [4–6]. In our previous studies, we found accurate robotic in-plane insertions of various types of needles in animal experiments [4, 5]. However, robotic out-of-plane insertions had not yet been evaluated. Unlike manual insertion, the robot allows users to set the needle at an exact predetermined angle before insertion and the needle posture during robotic insertion may be more stable. Thus, we hypothesized that accurate out-of-plane needle insertion could be achieved with the use of the robot. The aim of the present study is, therefore, to evaluate the accuracy of robotic out-of-plane needle insertion in phantom and animal experiments.

## Materials and methods

**Fig. 1:**
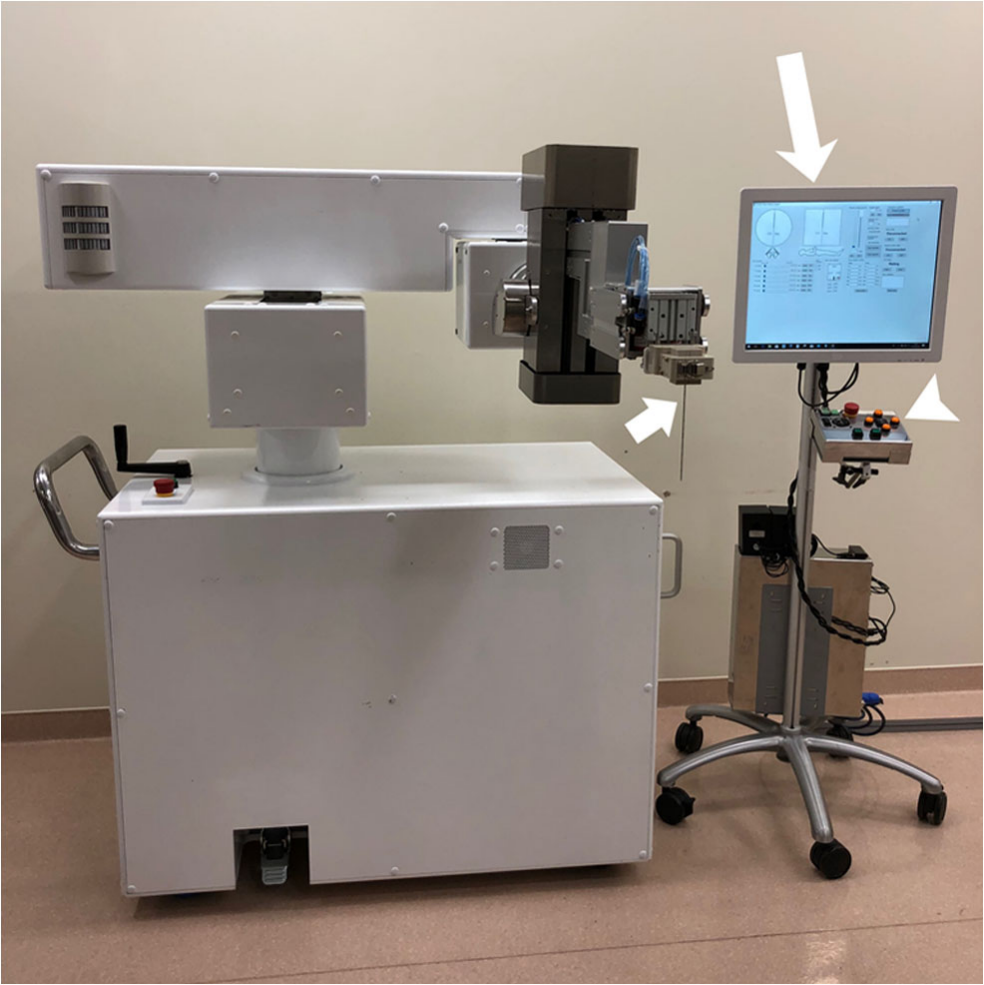
The robotic system. A robot (left) with 6 degrees of freedom and an interface (right) comprising a touch panel (long arrow) and a controller (arrowhead). The robot may be manipulated by either button operation of the controller or numerical inputs on software displayed on the touch panel. With the former technique, the robot moves while the buttons of the controller are manually pressed, whereas with the latter technique, the robot moves to a certain place semi-automatically after numerical inputs. In the present study, typically, alteration of needle angles for needle targeting and adjustment of needle orientation was performed by the latter, while needle insertion was done by the former. The needle (short arrow) is attached to a plastic needle holder at the end of a robot arm

The animal experiment was approved by the institutional animal care and use committee of our institution. The design of the robotic system (Zerobot, Medicalnet Okayama) used herein (Fig. 1) has been described previously [4–6]. Briefly, the system was designed by the authors (T.H., T. Kamegawa, and T. Matsuno) at Okayama University and then manufactured by Medicalnet Okayama. The robot has 6 degrees of freedom, and its tasks are to hold, locate, orient, and insert a needle under CT guidance. The needle is attached to a needle holder at the end of a robot arm. The robot may be manipulated by either button operation of the controller or numerical inputs on software displayed on the touch panel. With the former technique, the robot moves while the buttons of the controller are manually pressed, whereas with the latter technique, the robot moves to a certain place semi-automatically after numerical inputs. We had no role with the company that manufactured the robot.

### Phantom experiment

**Fig. 2:**
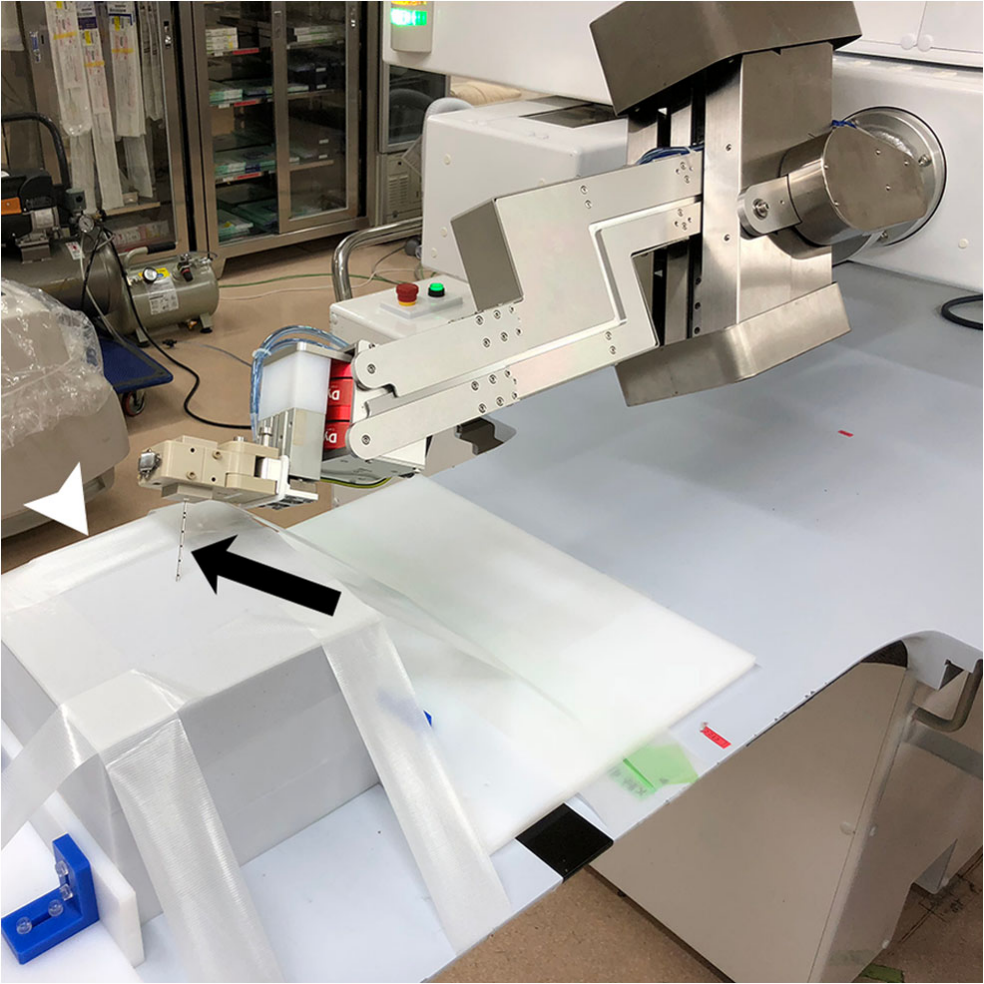
Robotic out-of-plane needle insertion in the phantom experiment. The needle (arrow) at the predetermined angles in the *XY* and *YZ* planes is inserted into a sponge phantom (arrowhead) fixed on the CT table

The phantom experiment was designed to evaluate the angle accuracy of robotic needle insertion, freehand manual insertion, and manual insertion guided by a smartphone (iPhone 6, Apple Inc.) application (SmartPuncture, Matsuyama Shimin Hospital). The endpoint was the angle errors in the *XY* and *YZ* planes (i.e., axial and sagittal CT planes, respectively). A 17-gauge biopsy introducer needle (TASK Laboratory) with six combinations of angles (Table 1) was inserted to a depth of approximately 8 cm into a melamine sponge [7, 8] fixed on the CT table (Fig. 2). For robotic insertions, the operator (T. Komaki, who had a 5-year experience in CT-guided interventions and a 3-year experience in manipulation of the robot) operated the robot to set the needle at predetermined angles, followed by insertion. In the manual group, freehand insertions (i.e., without guiding tools) were performed first. Then, the smartphone-guided manual insertions were performed with similar techniques as previously described [7]. Briefly, a planned angle in the *XY* plane was entered on the application. Then, a guideline with the angle was displayed on the screen. The tilted angle number of the smartphone against the direction of gravity, which corresponded to the angle in the *YZ* plane, was also displayed on the screen. The operator inserted the needle manually along the guideline while holding the smartphone with the planned angle in the *YZ* plane (Supplementary Fig. S1). Those manual insertions were performed by two operators (Y.M. and M.U. with 12-year and 11-year experiences, respectively, in CT-guided interventions). Needle insertion time was measured. CT scanning (Aquilion 64, Canon Medical Systems) was performed after each insertion.

**Table 1:** Needle Angles for Insertion in the Phantom Experiment

Needle angles (°)		No. of insertions				
		Robotic	Manual			
			Smartphone-guided		Freehand	
XY plane	YZ plane		Operator 1	Operator 2	Operator 1	Operator 2
40	-20	2	1	1	1	1
30	-30	2	1	1	1	1
20	-40	2	1	1	1	1
-20	20	2	1	1	1	1
-30	30	2	1	1	1	1
-40	40	2	1	1	1	1

The needle angle is defined as the angle between the perpendicular line and the needle on CT images. When the needle is oriented to the right and left side of the perpendicular line, the needle angle is expressed as a positive and negative value, respectively

The needle angle was measured in maximum intensity projections in the two planes at a CT console by a blinded author (S.O. with a 3-year experience in CT-guided interventions). The angle error was calculated as the difference between the predetermined angle and the angle after insertion. Then, the angle errors were compared between the robotic and smartphone-guided manual insertions. Using angle errors, the three-dimensional deviations of the needle tip at a depth of 8 cm were estimated (detailed calculation methods are provided in Appendix 1 in the Supplementary Material).

### Animal experiment

The animal experiment was performed to evaluate the in vivo distance accuracy of robotic out-of-plane insertion. The 17-gauge biopsy introducer needle (TASK Laboratory) was used for insertion in two female swine (weight, 56.7 kg and 54.3 kg) (management techniques are provided in Appendix 2 in the Supplementary Material). The hip muscle and the kidney were selected as locations for robotic insertion, as they allow for safe and adequately long out-of-plane needle tracts and evaluation of insertions both with and without respiratory motion. Before the experiment, 1.0-mm-diameter tungsten balls (Humanity) were placed into the locations as targets using an 18-gauge coaxial needle manually inserted with in-plane CT guidance. Depths of the targets from the skin ranged from 64.7 to 83.3 mm.

First, six insertions were made with three combinations of needle angles each into the hip muscle and the kidney (Table 2). These were performed without adjustments of the needle orientation during insertion, in order to evaluate the in vivo accuracy of methods similar to those adopted in the phantom experiment. Then, the same insertions were performed with adjustments during insertion, in order to confirm improved accuracy with adjustments. Insertions into the kidney were performed with breath hold by the ventilator, while insertions into the hip muscle were performed without breath hold.

**Table 2:** Needle Angles for Insertion in the Animal Experiment

Needle angles (°)		No. of insertions			
		With adjustments		Without adjustments	
XY plane	YZ plane	Hip muscle	Kidney	Hip muscle	Kidney
+30 ± 15	-30 ± 10	2	2	2	2
0 ± 15	-30 ± 10	2	2	2	2
-30 ± 15	-30 ± 10	2	2	2	2

The needle angle is defined as the angle between the perpendicular line and the needle on CT images. When the needle is oriented to the right and left side of the perpendicular line, the needle angle is expressed as a positive and negative value, respectively

**Fig. 3:**
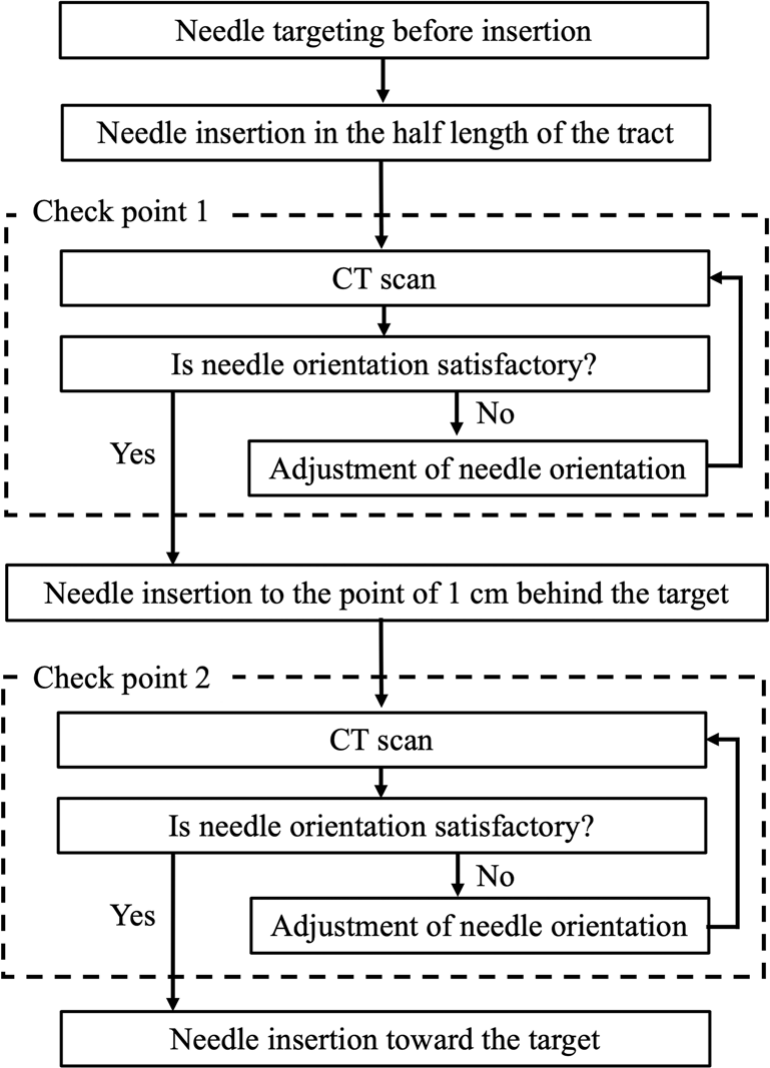
Techniques of needle insertion with adjustment in the animal experiment. Needle orientation is checked by CT scanning at two points: middle of the tract and 1 cm behind the target. Needle orientation is then evaluated in maximum intensity projections reconstructed from CT data in the *XY* and *YZ* planes. If the needle orientation is not satisfactory, it is adjusted until it becomes satisfactory

**Fig. 4:**
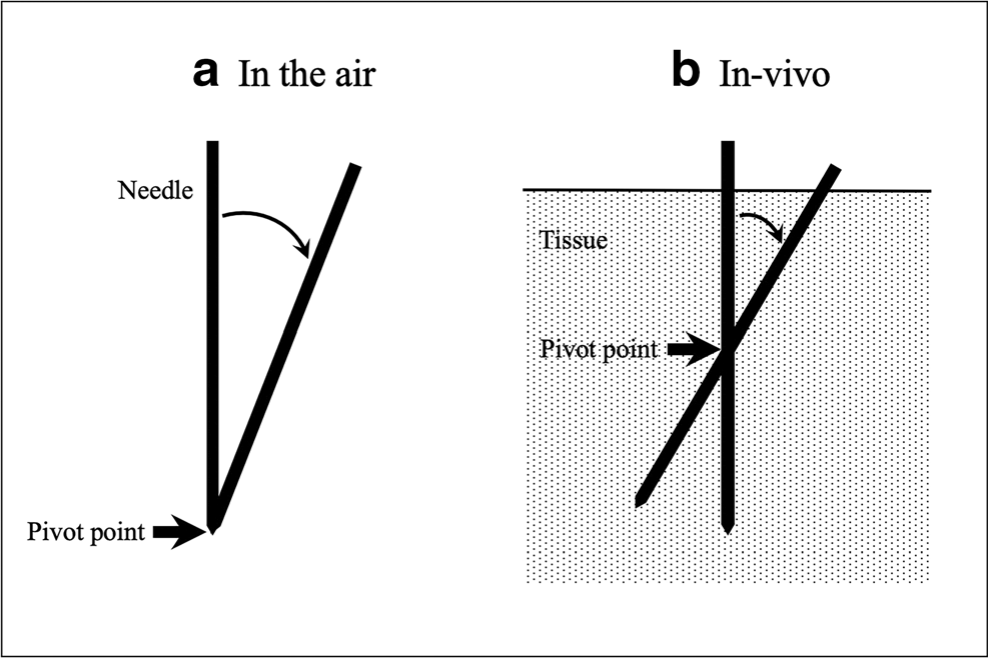
Schemata of the needle angle being changed by the robot in the air (**a**) and in vivo (**b**). The robot provides the remote-center-of-motion function, by which the needle angle is changed around its tip in the air. In vivo, however, the needle angle is changed as if a pivot point is at the approximately half length of needle in the tissue; this is attributable mainly to resistance of the tissue. Considering this characteristic needle behavior in vivo, the corrected needle angle in the planes may be calculated to compensate for the deviation between the ideal and actual needle angles

The robot was manipulated by the same author (T. Komaki) in the phantom experiment. CT scanning (Eminence STARGATE, Shimadzu Inc.) was performed to determine the starting point for insertion, the needle angles required, and the needle tract length. Subsequently, the needle was set with the angles and then its tip was moved to the starting point. Correct needle orientation was confirmed by CT scanning along with reconstructed three-dimensional CT images. The needle was then inserted till the whole tract length at once in the group without adjustment. In the group with adjustment, in contrast, the needle orientation was checked by CT scans at two time points (Fig. 3). The needle orientation was corrected as necessary, based on deviations between the ideal and actual needle angles and needle behavior during alteration of needle angles in vivo (Fig. 4). In both groups, needle insertion time, radiation exposure (i.e., tube current-time product and dose-length product) to the swine during insertion, and the number of CT scans and needle adjustments during insertion were recorded. CT scanning was performed after insertion.

Distance accuracy was evaluated on axial CT images of 0.5 mm thickness with 0.3-mm intervals using software (OsiriX, version 5.7.1, OsiriX Foundation) by an author (S.O.). First, whether the needle hit the target was evaluated on the reconstructed three-dimensional images. The distance accuracy was then determined by calculating the Euclidean distance between the target center and the needle tip, using the CT coordinates. Lateral and depth errors were further evaluated (Appendix 3 in the Supplementary Material). The actual length of needle tract and the amount of target movement during insertion (i.e., the distance between the target centers before and after insertion) were calculated, using the CT coordinates.

### Statistical analysis

In the phantom experiment, the number of needle insertions was calculated to verify the superiority of the robotic insertion compared to smartphone-guided manual insertion with respect to the angle accuracy. The absolute value of the mean difference in the angle errors between the two groups was estimated to be 1.0°, with a standard deviation of 0.5° in both groups. The correlation coefficient between the two planes (i.e., the *XY* and *YZ* planes) was hypothesized to be 0.3°. With an *α* value of 0.05 and a *β* value of 0.2, 12 insertions in each group were required.

Numerical variables were compared using a two-sided Student’s *t* test. A *p* value of < 0.05 was considered statistically significant. Statistical analysis was performed by an author (S.O.) using EZR software (EZR, version 1.33, Saitama Medical Center).

## Results

### Phantom experiment

The results of the phantom experiment are summarized in Table 3. The mean angle errors of the robotic, freehand manual, and smartphone-guided manual insertions were 0.4° (range, 0.0–1.6°), 7.0° (range, 1.0–17.1°), and 3.7° (range, 0.0–7.4°) in the *XY* plane and 0.6° (range, 0.0–1.2°), 6.3° (range, 1.8–12.1°), and 0.6° (range, 0.1–1.5°) in the *YZ* plane, respectively. Robotic insertions were significantly (*p* < 0.001) more accurate than smartphone-guided manual insertions in the *XY* plane. Robotic insertions resulted in significantly (*p* < 0.001) smaller predicted needle tip deviations (mean, 1.0 mm; range, 0.1–2.5 mm) than did smartphone-guided manual insertions (mean, 4.9 mm; range, 0.9–9.7 mm) at the insertion depth of 8 cm. Robotic insertions were significantly (*p* < 0.001) faster (mean, 5.0 s; range, 4.8–5.1 s) than smartphone-guided manual insertions (mean, 24.7 s; range, 13.8–43.3 s).

**Table 3:** Results of the phantom experiment

	Robotic	Manual		*p* value^a^
		Smartphone-guided	Freehand	
Needle insertion time (s)	5.0 ± 0.1 (4.8–5.1)	24.7 ± 8.1 (13.8–43.3)	4.4 ± 1.8 (3.0–9.4)	< 0.001
Needle insertion accuracy (°)				
*XY* plane	0.4 ± 0.4 (0.0–1.6)	3.7 ± 2.3 (0.0–7.4)	7.0 ± 5.7 (1.0–17.1)	< 0.001
*YZ* plane	0.6 ± 0.4 (0.0–1.2)	0.6 ± 0.4 (0.1–1.5)	6.3 ± 3.5 (1.8–12.1)	0.65
Predicted needle tip deviation at a depth of 8 cm (mm)	1.0 ± 0.7 (0.1–2.5)	4.9 ± 2.9 (0.9–9.7)	13.0 ± 7.0 (4.5–29.4)	< 0.001

Data are means ± standard deviations, with ranges in parentheses

^a^Comparison between robotic and manual smartphone-guided insertions with Student’s *t* test

### Animal experiment

The results of the animal experiment are summarized in Table 4. The mean tract length was 82.2 mm (range, 71.1–87.5 mm) and 82.5 mm (range, 69.9–92.0 mm) in the groups with and without adjustments, respectively. The needle appeared to hit the target in 4 of the 12 insertions without adjustment and in 11 of the 12 insertions with adjustment. The mean distance accuracy of the robotic needle insertions with adjustment was 2.5 mm (range, 0.9–3.8 mm) in the hip muscle and 2.4 mm (range, 1.9–3.3 mm) in the kidney, while that of robotic needle insertions without adjustment was 5.1 mm (range, 3.7–6.6 mm) in the hip muscle and 5.0 mm (range, 2.7–8.3 mm) in the kidney. Overall distance accuracy with adjustment (mean, 2.5 mm; range, 0.9–3.8 mm) was significantly (*p* < 0.001) better than that without adjustment (mean, 5.0; range, 2.7–8.3 mm). The results of lateral and depth errors are shown in Supplementary Table S1. Time for needle insertions with adjustment (mean, 716.9 s; range, 316–1851 s) was significantly (*p* < 0.001) longer than that without adjustment (mean, 14.4 s; range, 11–19 s), requiring the median of four CT scans and the median of two needle adjustments during insertion. The tube current-time product (mean, 7210.1 mAs; range, 3999–14,533 mAs) and dose-length product (mean, 998.3 mGy·cm; range, 553.8–2011.7 mGy·cm) during insertion in the group with adjustments were significantly (*p* < 0.001 for both) larger than the tube current-time product (mean, 1310.8 mAs; range, 1066–1333 mAs) and dose-length product (mean, 181.5 mGy·cm; range, 147.7–184.6 mGy·cm) during insertion in the group without adjustments.

**Table 4:** Results of the animal experiment

	Robotic insertion		*p* value
	With adjustment	Without adjustment	
Needle tract length (mm)^a^	82.2 ± 5.3 (71.1–87.5)	82.5 ± 8.0 (69.9–92.0)	0.903
No. of needle adjustments during insertion^b^	2 (0–5)	0 (0–0)	< 0.001
No. of CT scans during insertion^b^	4 (2–8)	0 (0–0)	< 0.001
Needle insertion time (s)^a^	716.9 ± 396.0 (316–1851)	14.4 ± 2.6 (11–19)	< 0.001
Distance of target movement (mm)^a^	4.6 ± 2.5 (1.2–11.2)	4.8 ± 0.8 (3.8–6.6)	0.776
Radiation exposure to swine during insertion			
Tube current-time product (mAs)^a^	7210.1 ± 2746.4 (3999–14,533)	1310.8 ± 77.1 (1066–1333)	< 0.001
Dose-length product (mGy·cm)^a^	998.3 ± 380.1 (553.8–2011.7)	181.5 ± 10.7 (147.7–184.6)	< 0.001
Needle insertion accuracy (mm)			
Hip muscle (*n* = 6)^a^	2.5 ± 1.0 (0.9–3.8)	5.1 ± 1.2 (3.7–6.6)	0.003
Kidney (*n* = 6)^a^	2.4 ± 0.5 (1.9–3.3)	5.0 ± 2.2 (2.7–8.3)	0.019
Total (*n* = 12)^a^	2.5 ± 0.8 (0.9–3.8)	5.0 ± 1.7 (2.7–8.3)	< 0.001

^a^Data are means ± standard deviations, with ranges in parentheses

^b^Data are medians, with ranges in parentheses

## Discussion

The present study was conducted to evaluate the accuracy of out-of-plane needle insertion using our robot; such an insertion is generally difficult to perform accurately by hand. The results showed that our robot achieved accurate out-of-plane needle insertions in the phantom and the animals, which may indicate more choices for selection of needle trajectories in clinical cases, possibly making the procedure safer and more effective. In particular, it is notable that junior staff and even residents may perform out-of-plane insertion with difficult trajectories using our robot.

Smartphone applications to assist needle insertion such as SmartPuncture and OncoGuide (National Institutes of Health) seem quite unique [7, 9]. A phantom study [7] indicated that the mean angle errors of smartphone-guided needle insertion were < 1.8° in the *XY* plane and < 4.1° in the *YZ* plane. The phantom experiment in the current study revealed that robotic out-of-plane needle insertion was significantly more accurate than smartphone-guided manual insertion. The advantages of the robot are that the needle angles required may be easily and accurately obtained by numerical inputs on the touch panel and the angles may be maintained during insertion.

Despite accurate needle angles in the phantom, the distance accuracy of robotic insertions in animals was limited to some extent if the needle orientation was not adjusted during insertion. This was attributed mainly to movement of targets and needle deviation during insertion [4, 5]. Although the distance accuracy of insertions without adjustment up to 8.3 mm might be acceptable for some interventional procedures (e.g., biopsy for lesions of ≥ 17 mm in diameter), more accurate insertion is usually required for lesions that are small and/or make contact with at-risk structures. To improve the accuracy, we adopted two check points to correct needle orientation. Needle deflection, which was more likely to occur by tissue displacement (especially the skin and subcutaneous tissue) during insertion, was mainly compensated at the half point of the tract. Target movement, which was more likely to occur when the needle tip was close to the target, was mainly compensated at the point of 1 cm behind the target. The two-step needle adjustment during insertion significantly improved the accuracy to a mean value of 2.5 mm. Such distance accuracy seems comparable to that of in-plane insertion with this robot in previous animal experiments [4, 5]. Notably, however, insertions with adjustment required greater radiation exposure as well as more time. Therefore, the necessity and appropriate number for needle adjustments should be determined individually based on the accuracy required in the case.

Other than our robot, several devices and robots to assist out-of-plane needle insertion have been evaluated. An electromagnetically guided system (IMACTIS) displays the needle path in real time on two-dimensional CT images reconstructed from preprocedural CT data [3, 10]. A phantom study [3] reported a median distance accuracy of 3.7 mm in out-of-plane trajectories using this navigation system. In addition, a prospective randomized clinical trial [10] demonstrated that the needle insertion accuracy was significantly improved with this navigation system, when compared to insertion with conventional CT guidance (median distance accuracy, 4.1 mm vs. 8.9 mm) in various clinical conditions including out-of-plane trajectory. Some robotic positioning systems have been commercialized, including iSYS (Kitzbuhel) [8, 11–13] and MAXIO (Perfint Healthcare) [14–17]. Unlike our robotic system, the task of those robots is confined to needle targeting (i.e., orientation of the needle) based on preprocedural CT data. Therefore, needle insertion must be manually performed by physicians. Accuracy of needle insertion including out-of-plane trajectory with these systems has been evaluated [8, 11–17]. For example, an animal study using MAXIO [15] demonstrated that the mean distance accuracy was 4.7 mm, which seems comparable to the insertion accuracy without adjustment in our animal experiment. The abovementioned electromagnetically guided system and robotic positioning systems are based on preprocedural CT data and therefore do not allow for a response to intraprocedural positional alteration (e.g., target movement and the patient’s body motion) and needle deviation. On the other hand, our robot enables response to intraprocedural alteration, which has the potential to improve insertion accuracy.

There were some limitations to our study. The type of the needle used, locations for needle insertion in swine, and combinations of needle angles tested were limited. Further, techniques of breath hold with the ventilator employed in the animal experiments were different from those in conscious patients. Therefore, it remains to be confirmed whether similar results can be obtained with other needles and angles at other locations in conscious clinical cases; this is an area requiring future research.

In conclusion, robotic CT-guided out-of-plane needle insertions were more accurate than smartphone-guided manual insertions in the phantom and were also quite accurate in the in vivo procedure, particularly with adjustment of needle orientation during insertion.

## Electronic supplementary material

ESM 1 (DOCX 4739 kb)

## Abbreviation

CT: Computed tomography
