# Supplementary material for: Robotic CT-guided out-of-plane needle insertion: comparison of angle accuracy with manual insertion in phantom and measurement of distance accuracy in animals
Source: Eur Radiol. 2019 Nov 26;30(3):1342–9. doi: 10.1007/s00330-019-06477-1 (PMC7033049; doi:10.1007/s00330-019-06477-1)
Supplement: Supplementary file 1 — (DOCX 4739 kb) [file 330_2019_6477_MOESM1_ESM.docx]

**Appendix 1: Detailed calculation methods of the three-dimensional deviations of the needle tip using angle errors in the phantom experiment**

First, $\theta_{x}$ is defined as a needle angle from the Y axis around the Z axis; $\theta_{z}$ is an angle from the Y axis around the X axis. In other words, $\theta_{x}$ is the projected posture of the needle onto the XY plane, and $\theta_{z}$ is that onto the YZ plane. Those actual needle angles are obtained from a CT volume image using application tools. Next, $e_{x},e_{y},{\mathrm{and} e}_{z}$ are defined as components of a unit vector, which is along the needle posture in 3-dimensional space. Based on the relation between the angle and unit vector,

$$\tan\theta_{x}=-\frac{e_{x}}{e_{y}} , (1)$$

$$\tan\theta_{z}=\frac{e_{z}}{e_{y}}, (2)$$

$${e_{x}}^{2}+{e_{y}}^{2}+{e_{z}}^{2}=1 (3)$$

are obtained. In accordance with right-handed screw rule, equation (1) has a negative sign on the right side. Finally, by rewriting the equations from (1) to (3),

$$e_{y}=\sqrt{\frac{1}{\left( 1+\text{tan}^{2}\theta_{x}+\text{tan}^{2}\theta_{z} \right)}\text{ }} (4)$$

$$e_{x}=-e_{y}\tan\theta_{x} (5)$$

$$e_{z}=e_{y}\tan\theta_{z} (6)$$

are obtained.

Once both the unit vector for the desired needle posture $e_{x}^{*},e_{y}^{*},{\mathrm{and}e}_{z}^{*}$ and that for the actual needle posture $e_{x}^{a},e_{y}^{a}, \mathrm{and}e_{z}^{a}$ are obtained by the above-mentioned equations, the angle offset between them $\phi$ can be calculated with the following equation:

$$\phi=\cos^{-1} \left( e_{x}^{*}e_{x}^{a}+e_{y}^{*}e_{y}^{a}+e_{z}^{*}e_{y}^{a} \right). (7)$$

Finally, $d$, the deviation of the needle tip from the desired state after insertion, can be estimated using

$$d=2L\sin\frac{\phi}{2}, (8)$$

where $L$ is the inserted needle length$.$

**Appendix 2: Management techniques of the animals**

Swine were sedated with intramuscular ketamine combined with medetomidine and midazolam. Atropine sulfate was also intramuscularly administered. General anesthesia was introduced and maintained using isoflurane with oxygen provided via an endotracheal tube. A ventilator controlled the aspiration of the animals. Vital parameters, including O2 saturation, pulse, blood pressure, end-tidal CO2, and body temperature were monitored throughout the procedure.

**Appendix 3: Lateral and depth errors in the animal experiment**

In addition to the Euclidean distance between the target center and the needle tip, lateral and depth errors were evaluated in the animal experiment. Lateral errors refer to the perpendicular distance between the target center and the needle path, while depth errors are measured as the longitudinal distance between the needle tip and the perpendicular axis at the target center [1]. The results of lateral and depth errors in the animal experiment are summarized in Supplementary Table S1.

**Reference**

1. Widmann G, Stoffner R, Sieb M, Bale R (2009) Target registration and target positioning errors in computer-assisted neurosurgery: proposal for a standardized reporting of error assessment. Int J Med Robot 5:355­365

**Supplementary Figure S1: Smartphone-guided manual needle insertion**

**
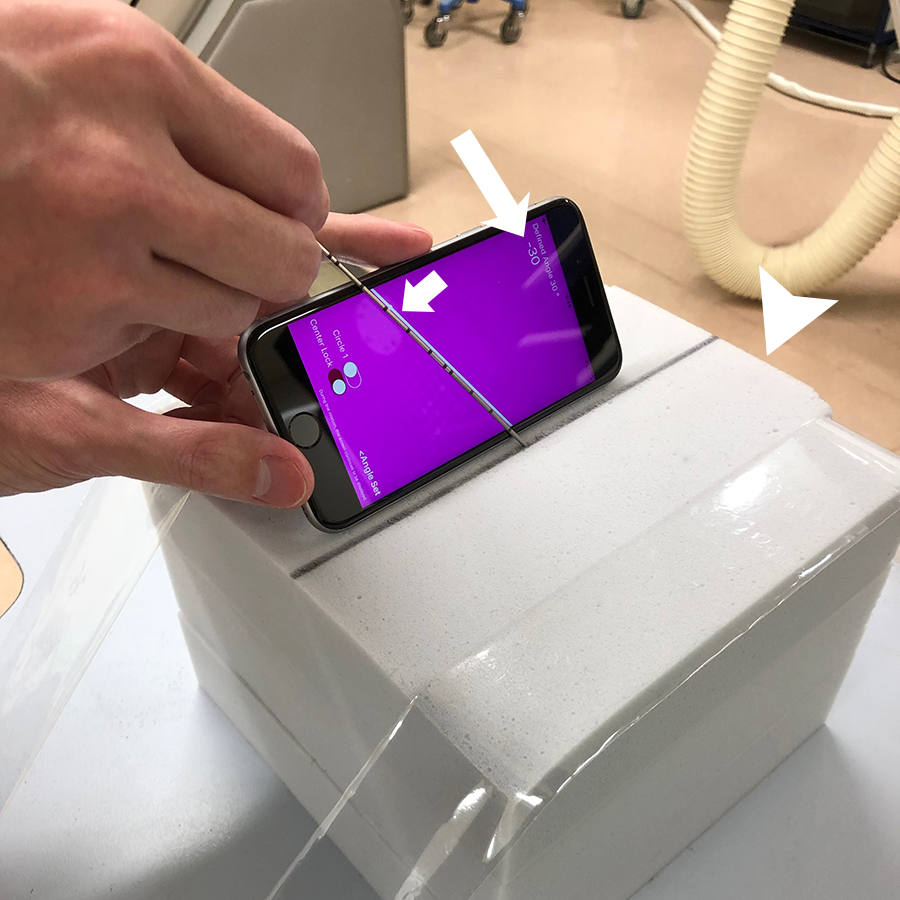
**

The smartphone application (SmartPuncture) is a guidance tool for manual needle insertion. By entering a planned angle in the XY plane on the application, a guideline (short arrow) with the angle is displayed on the screen. The tilted angle number of the smartphone against the direction of gravity is also displayed on the screen (long arrow), which corresponds to the angle in the YZ plane. The operator inserts the needle manually along the guideline, while holding the smartphone on a sponge phantom (arrowhead) with the planned angle in the YZ plane.

**Supplementary Table S1: Results of Lateral and Depth Errors in the Animal Experiment**

|  | | Robotic insertion | | *P* Value |
| --- | --- | --- | --- | --- |
|  |  | with adjustment | without adjustment |  |
| Needle insertion accuracy (mm) | |  |  |  |
| Lateral error | |  |  |  |
|  | Hip muscle (*n* = 6) | 2.2 ± 1.0 (0.5–3.5) | 5.0 ± 1.2 (3.7–6.6) | 0.001 |
|  | Kidney (*n* = 6) | 1.8 ± 1.0 (0.6–3.3) | 4.8 ± 2.3 (2.1–8.3) | 0.016 |
|  | Total (*n* = 12) | 2.0 ± 1.0 (0.5–3.5) | 4.9 ± 1.8 (2.1–8.3) | <0.001 |
| Depth error | |  |  |  |
|  | Hip muscle (*n* = 6) | 1.1 ± 0.8 (0.2–1.9) | 0.4 ± 0.5 (0.0–1.3) | 0.108 |
|  | Kidney (*n* = 6) | 1.2 ± 0.8 (0.3–2.0) | 1.0 ± 0.6 (0.2–1.8) | 0.729 |
|  | Total (*n* = 12) | 1.1 ± 0.8 (0.2–2.0) | 0.7 ± 0.6 (0.0–1.8) | 0.171 |
| Data are means ± standard deviations, with ranges in parentheses. | | | |  |
